# Why perceived organizational virtue matters: a psychological mechanism

**DOI:** 10.3389/fpsyg.2026.1788145

**Published:** 2026-04-16

**Authors:** Sung-Hoon Ko

**Affiliations:** Graduate School of Education, Kyonggi University, Suwon, Republic of Korea

**Keywords:** job performance, leader-member exchange, organizational virtue, positive psychological capital, social exchange theory

## Abstract

**Objective:**

This study investigates the mechanism linking employees’ perceived organizational virtue, leader–member exchange (LMX), and job performance. Specifically, this study examines the mediating role of LMX and the moderated mediation role of positive psychological capital (PsyCap) within this relationship.

**Methods:**

Structural equation modeling (SEM) and the PROCESS Macro were employed to test the proposed hypotheses. The analyses were conducted using a sample of employees in South Korea.

**Results:**

The results showed that organizational virtue was positively related to LMX, and that LMX, in turn, is positively associated with job performance. Furthermore, LMX mediated the positive relationship between organizational virtue and job performance. PsyCap moderated the mediated relationship between organizational virtue and job performance via LMX, operating as a moderator that strengthens the effect of LMX on job performance. Specifically, the mediating effect of LMX on the link between organizational virtue and job performance was stronger for employees with higher levels of PsyCap.

**Conclusion:**

These findings provide important theoretical implications by demonstrating that LMX and PsyCap jointly strengthen the positive relationship between organizational virtue and job performance. This study also provides practical implications by emphasizing that cultivating a virtuous organizational culture and developing employees’ PsyCap are vital for enhancing relational dynamics and employees’ job performance.

## 1 Introduction

Ethical considerations and psychological well-being have become increasingly important in organizational management as essential elements of sustainable organizational growth (Cameron et al., 2004; Di Fabio et al., 2023). It aligns with the United Nations’ 2030 Agenda, which frames sustainable development in terms of cultivating inclusive and ethically grounded organizational environments (United Nations, 2025). Organizational virtue, defined as the shared endorsement of values such as morality, fairness, trust, and respect among organizational members, can be indicative of an organization’s capacity to advance social sustainability at work (Rego et al., 2010). Empirical evidence further suggests that organizational virtue is associated with stronger affective commitment and greater organizational citizenship behavior among employees, vital for organizational performance and the development of sustainable workplaces (Podsakoff et al., 2000).

Recent research has increasingly conceptualized organizational virtue not merely as an ethical climate variable but as a strategic intangible asset that contributes to long-term resilience and adaptability (e.g., Di Fabio and Rosen, 2020; Chen W. et al., 2021). In particular, studies published within the past five years emphasize that virtue-based organizational environments foster psychological safety, collective efficacy, and prosocial motivation, which in turn enhance sustainable performance outcomes (Hannah et al., 2014; Jin et al., 2022). By integrating ethical climate theory with positive organizational scholarship, contemporary debates highlight the need to examine the multilevel mechanisms through which organizational virtue translates into measurable performance outcomes.

Despite these insights, empirical research examining the pathways through which organizational virtue facilitates sustainable job performance remains in its early stages. Enhancing both job performance and psychological well-being has been identified as an important condition for long-term organizational sustainability (Dimitriou and Papakostas, 2022; Hajar et al., 2022; Deci and Ryan, 2013). In this context, examining the psychological and relational processes through which organizational virtue is related to job performance holds significant theoretical and practical significance.

While prior studies have primarily focused on the direct effects of virtue on attitudinal outcomes, scholars have recently called for more process-oriented models that clarify the relational and psychological mechanisms underlying these effects (Newman et al., 2014; Babalola et al., 2022). Addressing this gap is essential to situating organizational virtue within broader debates concerning sustainable human resource management and socially responsible leadership.

Therefore, the present study is guided by the following research question:

RQ. How does employees’ perception of organizational virtue influence their job performance, and what roles do leader-member exchange (LMX) and positive psychological capital (PsyCap) play in this process?

Past research has demonstrated that LMX, which reflects mutual trust, respect, and high-quality communication between leaders and employees, is positively related to employees’ job satisfaction, organizational commitment, and performance (Graen and Uhl-Bien, 1995). High-quality LMX allows employees greater access to both instrumental resources and emotional support from their leaders, contributing to sustained levels of job performance (Liden et al., 1993). Moreover, organizational virtue enhances employees’ perceptions of fairness and trust in leadership, thereby supporting the development of high-quality LMX relationships (Rego et al., 2010), a relational condition that is critical for organizational sustainability.

More recent meta-analytic and empirical findings further indicate that LMX plays a pivotal mediating role in translating ethical and value-based organizational contexts into employee performance outcomes (Martin et al., 2016; Gottfredson et al., 2020). Emerging studies also suggest that in sustainability-oriented organizations, high-quality LMX relationships amplify employees’ sense of meaningful work and long-term commitment (Breevaart et al., 2014; Feng and Adams, 2023). These findings underscore the relevance of examining LMX as a central relational mechanism linking organizational virtue to performance.

PsyCap, which consists of self-efficacy, optimism, hope, and resilience (Luthans et al., 2006), provides employees with support in adapting to change and realizing their capabilities, thus contributing to enhanced performance (Stajkovic and Luthans, 1998). In particular, PsyCap functions as an internal personal resource that can enhance the quality of LMX (Avey et al., 2011). Prior research has shown that PsyCap enhances job performance and psychological well-being among employees (Avey et al., 2008, 2011; Dubey et al., 2020; Williams et al., 2015). In addition, David et al. (2024) showed that the relationship between PsyCap and job performance is mediated by employee well-being.

Recent scholarship has further positioned PsyCap within the framework of Conservation of Resources (COR) theory, emphasizing its role as a dynamic psychological resource that interacts with contextual resources such as leadership and organizational climate (Hobfoll, 1989; Liu et al., 2024). Studies conducted within the past five years indicate that employees with higher PsyCap are more capable of leveraging high-quality LMX relationships to sustain performance under uncertainty and environmental turbulence (Dai and Fang, 2023; Chen et al., 2021).

It is important to note that few studies have investigated the interrelationships among organizational virtue, PsyCap, leadership, and job satisfaction (Shahid and Muchiri, 2019; Di Fabio et al., 2023). These studies emphasize the important roles of organizational virtue and PsyCap in explaining positive organizational outcomes (Luthans, 2002). However, empirical examinations integrating organizational virtue, LMX, and PsyCap within a single moderated mediation framework remain limited, particularly from a sustainability perspective. Addressing this limitation responds to recent calls for integrative models that bridge ethical organizational contexts, relational leadership processes, and individual psychological resources (Agarwal and Farndale, 2017; Avolio et al., 2004). Thus, PsyCap may serve as a moderator in the pathway linking organizational virtue to job performance through LMX, by enhancing employees’ capacity to build and maintain high-quality interactions with leaders. However, empirical studies examining the pathways among organizational virtue, LMX, and job performance, particularly the moderating role of PsyCap in the process, remain scarce. Accordingly, the goals of this study are to:

1. Investigate the relationships among employees’ perceived organizational virtue, LMX, and job performance.
2. Explore the mediating role of LMX in the positive relationship between organizational virtue and job performance.
3. Assess the downstream moderated mediation effect of PsyCap, such that PsyCap moderates the effect of LMX on job performance within the indirect relationship between perceived organizational virtue and job performance.

This study advances the theory by proposing and empirically testing an integrated model that links organizational virtue, individual psychological resources (i.e., PsyCap), and leadership interactions (i.e., LMX) in explaining employees’ job performance from a sustainability perspective. In addition, this study contributes to the literature by empirically testing a moderated mediation process, demonstrating that PsyCap strengthens the effect of LMX on job performance within the indirect relationship between organizational virtue and job performance. Practically, the findings emphasize the strategic value of fostering organizational virtue and developing employees’ PsyCap as sustainable internal resources that enhance the effectiveness of LMX, supporting long-term organizational sustainability.

## 2 Hypotheses development

The present study integrates multiple theoretical perspectives in a complementary and hierarchical manner. Social exchange theory (Blau, 1964; Cropanzano and Mitchell, 2005) provides the overarching relational foundation, explaining how organizational virtue fosters reciprocal and trust-based leader–member exchange relationships. Within this relational context, role theory (Katz and Kahn, 1978) clarifies how high-quality LMX shapes employees’ role expectations and encourages expanded role behaviors beyond formally prescribed tasks. In this sense, social exchange theory explains the emergence of high-quality exchanges, whereas role theory explains how such exchanges translate into performance-enhancing behaviors.

Building upon this relational and role-based foundation, self-determination theory (Deci and Ryan, 2000; Ryan and Deci, 2020) provides a motivational explanation for how relational support embedded in high-quality LMX is internalized into autonomous motivation and proactive work engagement. At the same time, conservation of resources theory (Hobfoll, 1989; Halbesleben et al., 2014) offers a resource-based perspective suggesting that individuals with greater psychological resources are more capable of acquiring, mobilizing, and amplifying additional resources. Accordingly, psychological capital functions as a boundary condition that strengthens the translation of relational resources into performance outcomes. Rather than offering competing explanations, these theories operate at interconnected levels—relational (social exchange), structural-role (role theory), motivational (self-determination theory), and resource-based (conservation of resources theory)—collectively providing a coherent and multilevel explanation of how organizational virtue ultimately enhances job performance.

Overall, by integrating virtue-based organizational contexts, relational leadership dynamics, and individual psychological resources, this study responds to recent calls for more systematic and process-oriented explanations of sustainable job performance in contemporary organizations.

### 2.1 Organizational virtue and LMX

In the present study, the conceptualization of organizational virtuousness proposed by Cameron et al. (2004) is adopted. They define organizational virtuousness as the aggregate display of virtuous behaviors, processes, and cultures within organizations that embody moral excellence and contribute to human flourishing. Organizational virtuousness encompasses qualities such as compassion, forgiveness, integrity, trust, and optimism, and reflects not merely compliance with ethical standards but the presence of moral goodness at the collective level (Bright et al., 2006). Importantly, this construct is grounded in positive organizational scholarship and emphasizes the amplifying effects of virtuousness, whereby virtuous behaviors generate self-reinforcing upward spirals of positive outcomes.

Organizational virtuousness is conceptually distinct from ethical climate and ethical culture. Ethical climate refers to shared perceptions regarding what constitutes ethically appropriate behavior in an organization (Victor and Cullen, 1988), and ethical culture emphasizes formal and informal systems that promote ethical conduct (Treviño et al., 1998). In contrast, organizational virtuousness transcends rule-based norms and focuses on aspirational moral excellence and collective human strengths. It is concerned not only with preventing unethical behavior but with cultivating positive moral character within the organization.

The construct is also distinct from ethical leadership, which centers on the moral behaviors and influence processes of individual leaders (Junaidi, 2024). While ethical leadership may contribute to fostering a virtuous organization, it remains a leader-level construct. Organizational virtuousness, by contrast, represents a collective, system-level property embedded in organizational practices and culture.

Similarly, organizational virtuousness differs from perceived organizational support (Eisenberger et al., 1986), which reflects employees’ beliefs about the extent to which the organization values their contributions and cares about their well-being. Perceived organizational support is grounded in social exchange processes, whereas organizational virtuousness reflects a broader moral orientation that extends beyond reciprocal support and emphasizes collective flourishing (Settoon et al., 1996).

Thus, consistent with Cameron et al. (2004), the present study conceptualizes organizational virtuousness as a higher-order, collective construct that captures the moral character and positive essence of the organization. This delineation clarifies its distinct theoretical contribution and differentiates it from related ethical and support-based constructs.

Virtue provides individuals with a fulfilling life and a meaningful sense of purpose (Becker, 1992), a sense of purpose (Eisenberg and Spinrad, 2014), and relief from ambiguity and anxiety (Weiner, 1993, p. 127). It fosters resilience when facing adversity, leading to a meaningful life, greater happiness, and improved health-related outcomes (Myers, 2000; Ryff and Singer, 1998). At the organizational level, virtue means the organization’s aspiration to help employees realize their full potential. Organizational virtue reflects a state wherein employees embrace virtue and act in accordance with moral principles within organizations (Cameron et al., 2004).

Organizational virtue encourages employees to take an active interest in organizational affairs, take responsibility, and engage collaboratively at work (Fernando and Almeida, 2012). It enhances an employee’s job performance (Cameron et al., 2004) while also preventing the occurrence of negative work events (Erkmen and Esen, 2012). Specifically, managerial virtue improves employees’ performance and organizational citizenship behavior (Graham and Van Dyne, 2006). Moreover, organizational virtue even contributes to enhanced organizational profitability, reputation, and brand recognition (Fernando and Almeida, 2012). Hence, organizational virtue can contribute to the development of various positive characteristics within organizations, such as trust, integrity, fairness, compassion, forgiveness, gratitude, humility, hope, and optimism, because it reflects the ideals that employees and organizations seek to realize when performing at their full potential (Cameron et al., 2004). Specifically, organizational virtue is associated with attributes such as organizational health, social development, moral well-being, honesty, and tolerance, all of which enhance organizational culture and communication (Caza et al., 2004). Hence, organizations characterized by high levels of virtue are likely to be better equipped to handle challenges in the workplace (Cameron et al., 2003).

Caza et al. (2004) further emphasize that organizational virtue, when combined with ethical behavior, produces protective and reinforcing effects. Employees in virtuous organizations work in environments characterized by honesty, empathy, and tolerance, fostering a sense of respect among employees (Tsachouridi and Nikandrou, 2016). Virtuous managerial behavior also enhances employee performance, stimulates the generation of innovative ideas, and even empowers employees (Bertland, 2009). Therefore, both the virtuous actions of employees and their experiences of virtue at work contribute to the development of a positive work environment and enhance their performance.

According to LMX theory, leaders do not maintain identical relationships with all subordinates. Instead, each LMX is unique and differentiated (Graen and Scandura, 1987; Uhl-Bien and Maslyn, 2003). Leaders may develop high-quality exchange relationships with certain subordinates while maintaining low-quality relationships with others (Graen and Uhl-Bien, 1995). Graen and Uhl-Bien (1995) define high-quality LMX relationships as being grounded on mutual respect, trust, and a reciprocal commitment to providing ongoing support. High-quality LMX is developed by frequent and comfortable interactions between leaders and employees, and a sense of intimacy, whereas low-quality LMX may be characterized by misunderstanding and mutual distrust between leaders and subordinates (Graen and Cashman, 1975; Maslyn and Uhl-Bien, 2001).

Social exchange theory (Blau, 1964), which suggests that social behavior arises from social exchange interactions aiming to optimize benefits while reducing costs, can serve as a theoretical framework to explain the relationship between organizational virtue and LMX. Specifically, virtue can serve as contextual factors that influence these interpersonal exchanges. That is, when employees perceive that their organization consistently upholds virtues, such as fairness, integrity, and compassion, they are more likely to feel genuinely valued and respected by the organization. These positive perceptions can enhance employees’ sense of obligation to reciprocate toward not only the organization but also their leaders who represent the organization in daily interactions (Eisenberger et al., 2010; Rhoades and Eisenberger, 2002). More specifically, organizational virtue can serve as a macro-level resource increasing employees’ willingness to participate in high-quality social exchanges with their leaders. That is, employees may view their leaders as moral agents of a virtuous organization, thereby initiating reciprocal behaviors such as increased trust, loyalty, and open communication, which are core components of high LMX (Graen and Uhl-Bien, 1995). Therefore, I propose the following hypothesis:

Hypothesis 1. Organizational virtue is positively related to LMX.

### 2.2 LMX and job performance

Participants in an exchange relationship must fulfill the expectations placed on them according to the norm of reciprocity, and the exchange should be perceived as equal and fair (Blau, 1964). Employees with high-quality LMX not only feel a sense of obligation to perform their tasks well but may also feel a responsibility to behave in ways that can benefit their leaders (Graen and Scandura, 1987).

Building on contemporary developments in social exchange theory, recent studies suggest that high-quality LMX activates both obligation-based reciprocity and identity-based motivation processes (Graen and Uhl-Bien, 1995). Specifically, when employees perceive that their leaders invest trust and socio-emotional resources in them, they internalize relational expectations and align their self-concept with role performance standards. This relational identification strengthens intrinsic motivation and increases discretionary effort, thereby translating relational quality into measurable job performance outcomes.

Employees with high-quality relationships with their leaders experience smoother communication, gain increased attention, trust, and autonomy from their leaders, and are assigned more challenging tasks. These altogether can increase their attachment to the organization and motivate them to work harder (Liden et al., 1993). Supporting this, past studies on LMX have demonstrated that the quality of LMX enhances employees’ job satisfaction, promotion opportunities, performance, and organizational citizenship behavior (Gerstner and Day, 1997; Kamdar and Van Dyne, 2007; Mardanov et al., 2008).

Recent empirical evidence further clarifies that psychological empowerment and work engagement function as key mediating mechanisms linking LMX to job performance (Lee and Kim, 2024). High-quality LMX enhances employees’ perceptions of meaning, competence, and autonomy, which in turn increase engagement and proactive performance behaviors. Moreover, LMX has been shown to foster psychological safety, enabling employees to experiment, voice ideas, and adapt to changing task demands without fear of negative consequences (Park et al., 2022). These motivational and resource-based pathways provide a more fine-grained explanation of how LMX contributes to both task performance and adaptive performance.

In addition, employees with high-quality LMX are more likely to have frequent interactions with their leaders, developing mutual respect and trust. As a result, they are likely to be assigned more interesting and engaging tasks, greater responsibility, and authority (Liden et al., 1993; Graen and Uhl-Bien, 1995; Yukl and Michel, 2006). Supporting this, past studies showed that the level of LMX perceived by employees enhances role behaviors, innovative behaviors, and even helping behaviors (Gerstner and Day, 1997). Graen et al. (1982) also demonstrated that employees with high-quality LMX are more committed to their organizations and exhibit high job performance. In a similar vein, Hackett et al. (2003) and Shin et al. (2012) showed that LMX increases employees’ job performance.

Extending this line of reasoning, recent longitudinal research indicates that the relationship between LMX and job performance is reciprocal and dynamic over time (Yu et al., 2024). High initial levels of LMX predict subsequent improvements in performance, which in turn further strengthen the quality of leader–member relationships, creating a virtuous cycle. Furthermore, evidence from multi-sector samples demonstrates that LMX explains incremental variance in employee performance beyond other contemporary leadership styles, underscoring its distinct theoretical contribution (Audenaert et al., 2019).

Role theory further provides support for the positive relationship between LMX and employees’ job performance. High-quality LMX can provide employees with clearer and more expansive roles (Katz and Kahn, 1978), which contributes to improved performance. When clear expectations and autonomy are granted, employees perform their tasks more creatively and proactively. Recent research refines this argument by demonstrating that role clarity mediates the LMX–performance relationship, particularly in environments characterized by uncertainty and role ambiguity (Zaheer et al., 2025; Tasdemir et al., 2025). By reducing cognitive strain and role conflict, high-quality LMX allows employees to allocate attentional and emotional resources more efficiently toward goal-directed behavior. Additionally, contextual moderators such as organizational support climate have been found to strengthen the positive effects of LMX on performance, suggesting that relational quality operates most effectively within supportive organizational environments (Henderson and Jeong, 2024).

Ultimately, high-quality LMX can enhance employees’ job performance, which in turn contributes to the overall competitiveness of the organization. Hence, this study predicts the following:

Hypothesis 2. LMX is positively related to job performance.

### 2.3 Mediating role of LMX

Building upon the aforementioned discussion, this study proposes that LMX mediates the positive relationship between organizational virtue and job performance. Organizational virtue can serve as a basis for enhancing the LMX. In organizational cultures that emphasize virtue, a leader’s behaviors are likely to be ethical and empathetic, which fosters high-quality relationships with employees (Bertland, 2009). Indeed, Caza et al. (2004) suggested that organizational virtue and ethical behavior amplify the positive ripple effects within the organization, fostering a perception among employees that they are being respected, which, in turn, stimulates performance-oriented behaviors and can lead to improved job performance. Social exchange theory (Blau, 1964) further provides a useful framework to explain the basis of such relationships in that positive treatment from the organization or leader leads employees to reciprocate with voluntary contributions and responsible behavior. Therefore, organizational virtue can enhance LMX, which can result in improved job performance.

Similarly, according to the role theory, organizational virtue can provide employees with emotional stability and ethical standards, thus forming a positive and trust-based organizational culture (Cameron et al., 2004). Such a virtuous organizational environment could enhance the quality of LMX. Role theory further explains how the employees’ roles are shaped by expectations and norms (Katz and Kahn, 1978). In high-quality LMX relationships, employees are expected to exhibit expanded roles beyond their formally assigned roles, which leads to enhanced job performance (Ahn et al., 2024). In other words, organizational virtue enables employees to experience trust and respect in their relationship with leaders, thereby enhancing the quality of LMX. This high-quality LMX results in employees being perceived as more autonomous and proactive role performers, facilitating work behaviors that go beyond standard role expectations, which, in turn, improves their job performance. Therefore, LMX can function as a psychological and behavioral mediator in the positive relationship between organizational virtue and job performance (Baron and Kenny, 1986).

Hypothesis 3. LMX mediates the positive relationship between organizational virtue and job performance.

### 2.4 Moderating role of PsyCap

LMX theory argues that high-quality LMX facilitates reciprocal exchanges that enhance positive work attitudes and performance (Graen and Uhl-Bien, 1995). Within high-LMX dyads, employees receive increased support, recognition, and developmental opportunities, increasing their work motivation (Dulebohn et al., 2012). However, although organizational virtue may create an environment that is conducive to forming high-quality LMX, I argue that the extent to which LMX translates into job performance is likely to vary depending on employees’ psychological resources.

PsyCap represents an individual’s positive psychological state, enabling them to set challenging goals, persist in adversity, and stay motivated in the workplace (Luthans et al., 2006; Newman et al., 2014; Avey et al., 2006). Employees with high PsyCap are better equipped to translate their relational resources into performance because they possess stronger internal capacities to initiate, sustain, and amplify performance-enhancing behaviors. Prior research demonstrates that PsyCap strengthens individuals’ ability to benefit from supportive leadership and relational resources such as LMX (Story et al., 2013; Walumbwa et al., 2010). These findings suggest that PsyCap is not only an outcome of social exchanges but also an important psychological catalyst that affects the extent to which employees leverage LMX to increase their job performance (Luthans et al., 2005).

Self-determination theory explains that the translation of relational support into autonomous motivation can vary by individuals’ psychological resources (Deci and Ryan, 2000; Ryan and Deci, 2020). High PsyCap employees are more likely to internalize the positive cues delivered within high-quality LMX, such as autonomy support and competence feedback, and subsequently convert them into intrinsically motivated performance behaviors (Kudisch et al., 2006). On the other hand, employees with low PsyCap may underutilize the relational and emotional benefits of LMX, resulting in lower performance.

Conservation of resources theory further supports the above prediction. According to the theory, individuals with greater resources are likely to be more able to gain and leverage additional resources (Hobfoll, 1989; Halbesleben et al., 2014). LMX constitutes important social resources, but its performance-enhancing effect is strengthened when employees possess appropriate psychological resources, such as PsyCap. High PsyCap employees are likely to perceive LMX as an opportunity to expand their resources and to translate the relational support into performance-oriented behaviors (Avey et al., 2009). On the other hand, low-PsyCap employees may lack the motivational and cognitive capacity to translate LMX into performance, thus weakening the indirect effect of organizational virtue.

Hypothesis 4. The indirect effect of organizational virtue on job performance through LMX varies depending on employees’ levels of PsyCap. Specifically, for employees with high PsyCap, the effect of LMX on job performance is strengthened, thereby amplifying the indirect effect of organizational virtue; conversely, for employees with low PsyCap, this indirect effect is relatively weaker.

**Figure 1:**
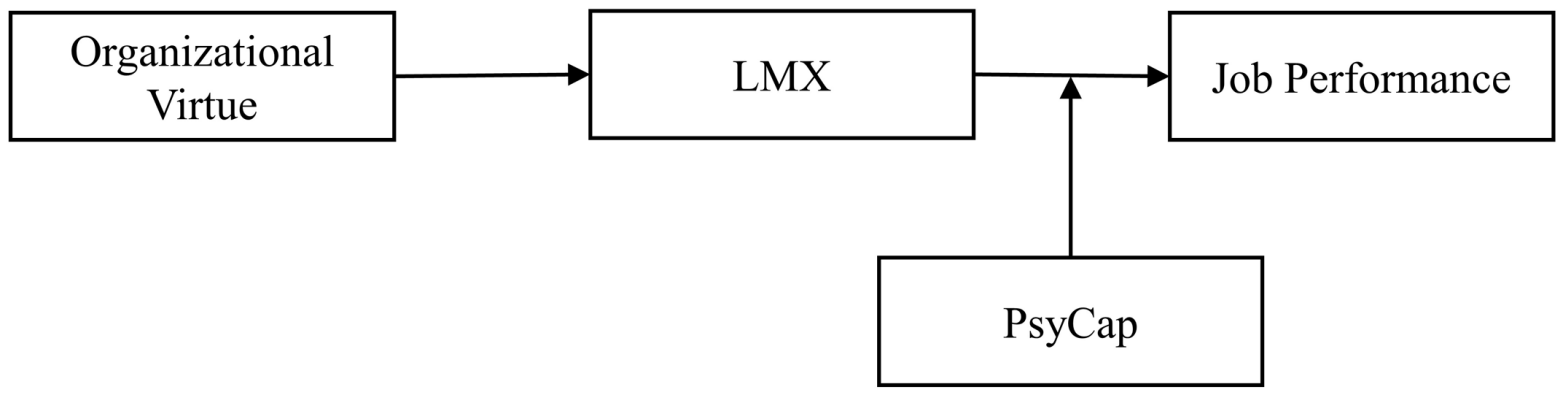
Research model.

The research model is presented in Figure 1.

## 3 Methods

### 3.1 Study participants and procedure

Data were collected through an online professional survey firm using a quota sampling strategy. To enhance demographic balance and reduce the risk of subgroup overrepresentation, quota controls were applied for gender (male/female) and age group (20s, 30s, 40s, 50s, and 60 or above). The survey firm was instructed to recruit participants so that each gender category and each age group would be equally represented in the final dataset. Importantly, the balanced demographic distribution reported in Table 1 reflects the pre-specified quota design implemented during data collection rather than post-hoc statistical adjustments. No weighting, re-sampling, or demographic calibration procedures were applied after data collection. The only exclusions involved removing careless or incomplete responses based on predefined data quality criteria. Although quota sampling may limit strict probabilistic generalizability, it enhances demographic comparability across groups and strengthens internal validity by minimizing demographic imbalance. This procedure allowed us to test the hypothesized relationships across evenly distributed demographic categories while maintaining transparency regarding sampling constraints.

**Table 1:** Frequency analysis.

Category	Item	Frequency	%
Gender	Male	160	50.0
	Female	160	50.0
Age group	20s	64	20.0
	30s	64	20.0
	40s	64	20.0
	50s	64	20.0
	60 or older	64	20.0
Education level	Enrolled in an undergraduate program	12	3.8
	Bachelor’s degree completed	253	79.1
	Enrolled in a master’s program	1	0.3
	Master’s degree completed	44	13.8
	Enrolled in a doctoral program or above	10	3.1
Years of service	1 ~ 4 years	139	43.4
	5 ~ 9 years	75	23.4
	10 ~ 14 years	32	10.0
	15–19 years	34	10.6
	20 years or more	40	12.5

A total of 391 employees participated in the survey. After excluding 71 cases due to careless or incomplete responses, a final sample of 320 employees was retained for the analysis. The survey targeted full-time employees working in team-based settings. The demographic characteristics of the final sample are summarized in Table 1. Regarding gender, 50.0% (160 individuals) were male, and 50.0% (160 individuals) were female. In terms of age, the distribution was evenly spread across groups, with 20.0% (64 individuals) in their 20s, 20.0% (64 individuals) in their 30s, 20.0% (64 individuals) in their 40s, 20.0% (64 individuals) in their 50s, and 20.0% (64 individuals) aged 60 or older. With respect to educational background, 3.8% (12 individuals) were currently enrolled in a university program, 79.1% (253 individuals) had completed a four-year university de-gree, 0.3% (1 individual) were enrolled in a graduate-level program, 13.8% (44 individuals) had completed a graduate-level program, and 3.1% (10 individuals) were enrolled in a doctoral-level program or higher. Tenure information indicated that 43.4% (139 individuals) had between 1 and 4 years of work experience, 23.4% (75 individuals) had 5 to 9 years, 10.0% (32 individuals) had 10 to 14 years, 10.6% (34 individuals) had 15 to 19 years, and 12.5% (40 individuals) had 20 years or more.

Taken together, these characteristics reflect a diverse set of employees across age, tenure, and educational levels, suitable for examining the hypothesized relationships among organizational virtue, LMX, and job performance.

### 3.2 Measures

All constructs were measured using a five-point Likert scale ranging from 1 (strongly disagree) to 5 (strongly agree). The coding scheme was consistent across all measures, with higher scores indicating higher levels of the respective construct. No reverse-coded items were included in the survey instrument. Consequently, no reverse scoring procedures were applied during data processing. All analyses were conducted using the original item coding.

*Organizational virtue* was measured by adapting 13 items by Cameron et al. (2004) to fit the context of this study. Example items include, “In our organization, members are committed not only to performing their tasks well but also to acting virtuously,” and “Our organization can be described as a moral and respectable company.” The reliability of the organizational virtue, as indicated by Cronbach’s alpha, was 0.944.

To measure *LMX*, the LMX scale, which consists of 12 items, was adopted from Liden and Maslyn (1998). The scale consists of four dimensions: affect, loyalty, contribution, and professional respect. Sample items include, “I admire my supervisor’s professional competence,” and “I go beyond what is formally required to support my supervisor.” Cronbach’s *α* was 0.949.

To measure *PsyCap*, I employed the 24 items by Luthans and Youssef (2007). Sample items include, “When faced with challenges at work, I can think of many ways to overcome them,” and “When experiencing setbacks and feelings of despair in the workplace, I can easily recover and move forward.” Cronbach’s alpha coefficient for PsyCap was *α* = 0.948.

*Job performance* was assessed using 5 items by Williams and Anderson (1994). Sample items include “I adequately complete assigned duties,” and “I fulfill responsibilities specified in the job description.” The reliability coefficient of the job performance scale, as indicated by Cronbach’s alpha, was 0.893.

## 4 Results

### 4.1 Confirmatory factor analysis (CFA)

To assess the adequacy of the measurement model, a CFA was conducted using maximum likelihood estimation (Kline, 2016; Arbuckle et al., 2016). Organizational Virtue and Job Performance were specified as first-order latent constructs. Consistent with their theoretical conceptualizations, LMX and Psychological Capital (PsyCap) were modeled as second-order latent constructs. Specifically, LMX was represented by four first-order dimensions—affect, loyalty, contribution, and professional respect (Liden and Maslyn, 1998)—whereas PsyCap was modeled as a higher-order construct comprising hope, efficacy, resilience, and optimism (Luthans et al., 2006; Peterson and Luthans, 2003).

The overall measurement model demonstrated an acceptable fit to the data: χ^2^(1430) = 2454.731, *p* < 0.001, CFI = 0.918, TLI = 0.911, RMSEA = 0.047, and RMR = 0.044. These indices meet or exceed commonly recommended thresholds (CFI and TLI ≥ 0.90; RMSEA ≤ 0.08) suggested by Hu and Bentler (1999) and Hair et al. (2010), indicating satisfactory model fit (Browne and Cudeck, 1992).

All standardized factor loadings were statistically significant at *p* < 0.001. The magnitude of factor loadings ranged from 0.712 to 0.861 for Organizational Virtue, 0.707 to 0.871 for LMX, 0.647 to 0.889 for PsyCap, and 0.716 to 0.824 for Job Performance. These loading magnitudes exceed the recommended minimum threshold of 0.50 (Hair et al., 2010), supporting indicator reliability.

Composite reliability (CR) values were 0.937 for Organizational Virtue, 0.936 for LMX, 0.944 for PsyCap, and 0.906 for Job Performance, all surpassing the 0.70 benchmark for internal consistency reliability (Fornell and Larcker, 1981; Hair et al., 2010). Average variance extracted (AVE) values ranged from 0.608 to 0.652, exceeding the 0.50 criterion and indicating adequate convergent validity (Fornell and Larcker, 1981). CFA results are presented in Table 2.

**Table 2:** Confirmatory factor analysis result.

Construct	Items	*λ*	SE	CR	AVE	Cronbach’s *⍺*	C.R
Organizational virtue	Virtue1	0.812	-	-	0.634	0.944	0.924
	Virtue2	0.794	0.074	13.506			
	Virtue3	0.755	0.085	13.069			
	Virtue4	0.797	0.085	13.851			
	Virtue5	0.720	0.091	12.539			
	Virtue6	0.841	0.091	14.522			
	Virtue7	0.809	0.088	13.978			
	Virtue8	0.822	0.082	14.298			
	Virtue9	0.754	0.088	13.074			
	Virtue10	0.861	0.096	11.420			
	Virtue11	0.801	0.091	10.383			
	Virtue12	0.712	0.090	12.314			
	Virtue13	0.854	0.087	13.034			
LMX	LMX1	0.754	-	-	0.652	0.949	0.937
	LMX2	0.864	0.059	18.113			
	LMX3	0.749	0.055	17.742			
	LMX4	0.871	0.069	16.643			
	LMX5	0.825	0.069	15.555			
	LMX6	0.838	0.068	15.940			
	LMX7	0.864	0.063	14.222			
	LMX8	0.707	0.063	13.014			
	LMX9	0.771	0.060	10.321			
	LMX10	0.812	0.064	15.274			
	LMX11	0.810	0.066	15.213			
	LMX12	0.804	0.072	15.095			
PsyCap	Psy1	0.742	-	-	0.613	948	0.914
	Psy2	0.771	0.110	10.759			
	Psy3	0.778	0.109	10.858			
	Psy4	0.699	0.099	10.544			
	Psy5	0.706	0.118	11.349			
	Psy6	0.734	0.090	8.795			
	Psy7	0.776	0.092	10.002			
	Psy8	0.781	0.093	10.829			
	Psy9	0.862	0.093	10.655			
	Psy10	0.736	0.109	10.340			
	Psy11	0.851	0.091	10.504			
	Psy12	0.889	0.097	11.024			
	Psy13	0.882	0.107	10.921			
	Psy14	0.722	0.099	11.454			
	Psy15	0.825	0.101	10.118			
	Psy16	0.647	0.112	10.501			
	Psy17	0.854	0.099	10.546			
	Psy18	0.651	0.098	10.483			
	Psy19	0.859	0.108	10.641			
	Psy20	0.705	0.099	11.237			
	Psy21	0.815	0.102	10.039			
	Psy22	0.752	0.101	10.510			
	Psy23	0.862	0.097	10.646			
	Psy24	0.816	0.102	10.005			
Job performance	JP1	0.814	-	-	0.608	0.893	0.896
	JP2	0.767	0.090	13.120			
	JP3	0.824	0.096	12.386			
	JP4	0.716	0.099	12.251			
	JP5	0.774	0.096	13.233			
	JP6	0.755	0.098	12.967			
	JP7	0.804	0.111	12.031			

χ^2^ = 2454.731 (df = 1,430, *p* = 0.000); CFI = 0.918; TLI = 0.911; IFI = 0.918; RMSEA = 0.047; RMR = 0.044.

All factor loadings are significant (*p* < 0.001). Virtue, organizational virtue; Psy, positive psychological capital, JP, job performance.

Taken together, these findings provide strong evidence for the reliability and convergent validity of the measurement model and support the appropriateness of modeling LMX and PsyCap as second-order latent constructs consistent with prior theoretical and empirical research.

### 4.2 Research model

Construct means, standard deviations, and correlations are presented in Table 3. To assess potential multicollinearity issues among the latent variables, Pearson correlation analyses were conducted. As shown in Table 3, the Pearson correlation coefficients ranged from 0.290 to 0.516 (*p* < 0.01), which are well below the generally accepted threshold for multicollinearity concerns (±0.80; Hair et al., 2010), indicating that multicollinearity is not a significant issue in the proposed research model.

**Table 3:** Construct means, standard deviations, and correlations.

Variables	Mean	SD	1	2	3	4
1. Organizational virtue	3.583	0.657	*0.796*			
2. LMX	3.552	0.700	0.515^**^	*0.807*		
3. PsyCap	3.622	0.525	0.433^**^	0.516^**^	*0.783*	
4. Job performance	3.973	0.541	0.290^**^	0.314^**^	0.480^**^	*0.780*

*p < 0.05; **p < 0.01; ***p < 0.001.

Furthermore, both the factor loadings and standardized factor loadings for each measurement item exceeded the traditionally recommended threshold of 0.50 (Fornell and Larcker, 1981; Bliese, 2000). Composite reliability (CR) values for all latent variables were above 0.70, demonstrating satisfactory internal consistency and reliability across the measurement items (Nunnally and Bernstein, 1994). In addition, the average variance extracted (AVE) values met or exceeded the recommended criterion of 0.60, with the square roots of AVE ranging from 0.776 to 0.807, thereby confirming adequate convergent validity for all constructs (Fornell and Larcker, 1981).

### 4.3 Hypotheses testing

In the present study, Hypotheses 1 and 2 were tested using structural equation modeling (SEM) with AMOS 27.0 software (Collier, 2020). SEM was employed to examine the structural relationships among the latent constructs based on the validated measurement model. Hypotheses 3 and 4, which involved mediation and moderated mediation effects, were tested using PROCESS Macro. This analytical approach enabled the estimation of indirect and conditional indirect effects through bootstrapping procedures (Hayes, 2013).

Although structural equation modeling (SEM) was used to validate the measurement model and test the primary structural relationships, mediation and moderated mediation were examined using PROCESS Macro (Hayes, 2018). PROCESS applies bias-corrected bootstrapping to estimate indirect and conditional indirect effects, which is widely recommended due to its higher statistical power and fewer distributional assumptions compared to traditional methods (Preacher and Hayes, 2008; Zhao et al., 2010).

Although SEM can estimate indirect effects, testing moderated mediation in covariance-based SEM requires latent interaction modeling through product-indicator approaches, which substantially increases model complexity and may cause estimation difficulties. In this study, construct validity was first established through SEM to minimize measurement error, and PROCESS was then applied to composite scores to estimate conditional indirect effects. Because the regression-based indirect effect in PROCESS is mathematically equivalent to that obtained from a path model using observed composites, additional SEM bootstrapping was considered unlikely to yield substantively different conclusions.

Hypothesis 1 proposed that organizational virtue would have a positive relationship with LMX. As shown in Table 4, the path coefficient from organizational virtue to LMX was *b* = 0.655, with a standard error of 0.047 and a critical ratio (CR) of 13.929 (*p* < 0.001), thus providing support for Hypothesis 1. It is consistent with prior research showing that a virtuous organizational climate increases LMX (Fredrickson, 2001; Cameron et al., 2004).

**Table 4:** Path analysis results.

Hypothesis	Path	*b*	SE	CR	*p*-value
Hypothesis 1	Organizational virtue → LMX	0.655	0.047	13.929	0.001
Hypothesis 2	LMX → Job performance	0.319	0.039	8.112	0.001

Hypothesis 2 suggested that LMX would have a positive relationship with job performance. The path coefficient from LMX to job performance was b = 0.319, with a standard error of 0.039 and a CR of 8.112 (*p* < 0.001), supporting Hypothesis 2. It aligns with previous research, which showed that high-quality LMX relationships enhance an employee’s job performance (Dulebohn et al., 2012).

To test the mediation hypothesis (Hypothesis 3), the bootstrapping method was employed using the PROCESS Macro (Hayes, 2018). Bootstrapping allows for the estimation of confidence intervals for indirect effects through repeated resampling, thereby providing a more robust assessment of the statistical significance of mediation effects (Preacher and Hayes, 2004, 2008. In the present study, the mediation hypothesis was examined using PRO-CESS Macro Model 4 with 5,000 bootstrap samples.

Although organizational virtuousness is theoretically conceptualized as a macro-level construct (Cameron et al., 2004), the present study focuses on employees’ individual perceptions of organizational virtuousness. Prior methodological research suggests that constructs originating at higher levels may be examined at the individual level when the focal mechanism concerns individual perceptions rather than objective organizational characteristics (Klein et al., 1994; Chan, 1998). Accordingly, organizational virtuousness was measured using individual survey responses and treated as a perceptual construct reflecting employees’ psychological experiences.

Because the dataset did not contain a sufficient number of higher-level organizational units to support reliable multilevel structural equation modeling, the hypotheses were tested at the individual level. Hypotheses 1 and 2 were examined using structural equation modeling (SEM) with AMOS 27.0 (Collier, 2020), whereas Hypotheses 3 and 4 were tested using PROCESS Macro with bootstrapping procedures to estimate mediation and moderated mediation effects.

The results indicated that organizational virtue did not have a statistically significant direct effect on job performance (*b* = 0.046, SE = 0.053, *p* > 0.05), suggesting that organizational virtue does not directly enhance employees’ job performance. However, the bootstrapping analysis revealed a significant indirect effect of organizational virtue on job performance through LMX. Specifically, the indirect effect was 0.191 with a 95% bootstrap confidence interval of [0.120, 0.270]. Because zero was not included in this confidence interval, the indirect effect was statistically significant (Hayes, 2018). These findings suggest that LMX serves as an important mechanism through which organizational virtue influences employees’ job performance. In other words, LMX partially mediates the relationship between organizational virtue and job performance. Accordingly, the results indicate that organizational virtue contributes to job performance both directly (in the absence of the mediator) and indirectly through LMX, highlighting the mediating role of high-quality leader–member exchange in translating organizational virtue into improved employee performance. This result supports Hypothesis 3 and underscores the critical role of high-quality LMX as a mechanism through which organizational virtue translates into improved employee performance. The detailed results of the direct and indirect effects are presented in Table 5.

**Table 5:** Direct and indirect effects of organizational virtue on job performance.

Effect type	Effect size	SE	*p*-value/95% CI
Direct effect			
OV → JP	0.046	0.053	>0.05
Indirect effect			
OV → LMX → JP	0.191	0.038	[0.120, 0.270] (95% CI)

OV, organizational virtue; LMX, leader-member exchange; JP, job performance.

To examine whether the mediating effect of LMX in the relationship between organizational virtue and job performance is moderated by PsyCap, this study employed PROCESS Macro Model 14 (Hayes, 2018). The results of the moderated mediation analysis are presented in Tables 6–8. As shown in Table 6, when the mediating variable was not included in the model, organizational virtue had a significant direct effect on job performance (*b* = 0.112, SE = 0.043, *t* = 2.598, *p* < 0.01), with a 95% confidence interval of [0.027, 0.197]. This result indicates that organizational virtue is positively associated with job performance in the absence of the mediator.

**Table 6:** Direct effect of organizational virtue on job performance (without mediation).

Effect	SE	*t*	*p*	LLCI	ULCI
0.112	0.043	2.598	< 0.01	0.027	0.197

**Table 7:** Conditional indirect effect of organizational virtue on job performance via LMX at different levels of PsyCap.

PsyCap level	Effect	Boot SE	Boot LLCI	Boot ULCI
-1 SD	3.097	0.042	0.012	0.042
Mean	3.622	0.036	0.052	0.089
+1 SD	4.148	0.041	0.004	0.166

PsyCap levels represent conditional values at −1 standard deviation (low level), the mean (moderate level), and +1 standard deviation (high level). Boot SE, bootstrap standard error; Boot LLCI, lower limit of the bootstrap confidence interval; Boot ULCI, upper limit of the bootstrap confidence interval.

**Table 8:** Index of moderated mediation.

Moderator	Index	Boot SE	Boot LLCI	Boot ULCI
PsyCap	0.121	0.040	0.041	0.198

Table 7 presents the conditional indirect effects of organizational virtue on job performance through LMX at different levels of PsyCap. The results showed that the indirect effect was statistically significant across all levels of PsyCap, as none of the bootstrap confidence intervals included zero. Specifically, when PsyCap was low, the indirect effect was 0.097 [Boot SE = 0.042, Boot CI (0.012, 0.042)]. At the mean level of PsyCap, the indirect effect increased to 0.122 [Boot SE = 0.036, Boot CI (0.052, 0.089)]. When PsyCap was high, the indirect effect further increased to 0.148 [Boot SE = 0.041, Boot CI (0.004, 0.166)]. These findings indicate that the mediating role of LMX becomes stronger as PsyCap increases.

Furthermore, the significance of the moderated mediation effect was confirmed by the index of moderated mediation presented in Table 8. The index value was 0.121 (Boot SE = 0.040), with a 95% bootstrap confidence interval of [0.041, 0.198]. Because zero was not included in the confidence interval, the index of moderated mediation was statistically significant (Hayes, 2018). This result demonstrates that PsyCap significantly moderates the indirect effect of organizational virtue on job performance via LMX.

These findings suggest that PsyCap functions as a critical personal resource (Luthans and Youssef, 2007) that amplifies the positive impact of organizational virtue on job performance (Luthans et al., 2007a) by strengthening the quality of LMX. Employees with higher levels of PsyCap are more likely to perceive virtuous organizational environments positively and to develop stronger LMX relationships, which in turn lead to enhanced job performance. Therefore, Hypothesis 4 was supported.

### 4.4 Common method variance testing

Because all variables in this study were measured using self-reported instruments, the potential influence of common method variance (CMV) was carefully evaluated (Podsakoff et al., 2003; Williams et al., 1989. I adopted the unmeasured latent method factor technique originally proposed by Williams and Anderson (1994) and further systematized by Podsakoff et al. (2003). Following established procedures, I estimated two nested models using robust maximum likelihood estimation (MLR). The baseline model included only the theoretically specified latent constructs, whereas the comparison model additionally incorporated an unmeasured latent method factor with all items loading onto both their theoretical constructs and the method factor.

The results are presented in Table 9. The baseline measurement model demonstrated acceptable fit to the data: χ^2^ = 2454.731 (df = 1,430, *p* < 0.001), CFI = 0.918, TLI = 0.911, IFI = 0.918, RMSEA = 0.047, and χ^2^/df = 1.716, indicating satisfactory model fit according to conventional criteria (Browne and Cudeck, 1993; Hu and Bentler, 1999). After introducing the latent method factor, model fit improved: χ^2^ = 2109.219 (df = 1,374, *p* < 0.001), CFI = 0.941, TLI = 0.934, IFI = 0.942, RMSEA = 0.041, and χ^2^/df = 1.535.

**Table 9:** Analysis of common method bias.

Model	χ^2^	df	*p*-value	χ^2^/df	RMSEA	CFI	IFI	TLI
Measurement model	2454.731	1,430	< 0.001	1.716	0.047	0.918	0.918	0.911
Controlled model	2109.219	1,374	< 0.001	1.535	0.041	0.941	0.942	0.934
Stepwise analysis	⊿	⊿df		Accepted model				
M.M-C.M	345.512	56	>0.05	Measurement model				

Because robust maximum likelihood estimation was used, the chi-square difference test was evaluated using the scaled chi-square difference testing procedure (Satorra–Bentler correction). Importantly, when robust corrections are applied, the difference between raw chi-square values does not follow a standard chi-square distribution and must be assessed using the corrected scaling factor. The robust scaled chi-square difference test yielded Δχ^2^ (56) = 345.512, *p* > 0.05. Thus, the improvement in model fit attributable to the addition of the method factor was not statistically significant after applying the appropriate correction.

Moreover, changes in practical fit indices were minimal and in the direction of marginal improvement (ΔCFI = 0.023; ΔRMSEA = −0.006). Inspection of standardized factor loadings further indicated that the inclusion of the method factor did not meaningfully alter the magnitude or significance of the substantive path coefficients. Consistent with the recommendations of Philip M. Podsakoff et al. (2003) and MacKenzie and Podsakoff (2012), these results suggest that common method bias is unlikely to pose a serious threat to the validity of the findings.

To further establish the robustness of the measurement structure, I compared the hypothesized multi-factor model against several theoretically plausible alternative models. First, a single-factor model in which all measurement items were constrained to load onto a single latent construct was estimated. This model demonstrated poor fit to the data (χ^2^ = 6,982.417, df = 1,485, CFI = 0.612, TLI = 0.584, RMSEA = 0.104), substantially worse than the hypothesized multi-factor model. These results indicate that collapsing all indicators into a single latent construct fails to adequately represent the underlying factor structure of the data. Second, additional competing models were tested by combining conceptually related constructs into fewer higher-order factors. These alternative specifications consistently yielded inferior fit indices relative to the proposed measurement model. The hypothesized multi-factor measurement model demonstrated significantly better fit than all alternative models, supporting discriminant validity among constructs and providing further evidence against substantial common method contamination (Campbell and Fiske, 1959).

As a supplementary diagnostic procedure, Harman’s single-factor test was conducted (Podsakoff et al., 2003). An exploratory factor analysis revealed that the first unrotated factor accounted for less than 40% of the total variance, below the commonly suggested threshold for serious method bias (Harman, 1976). Although this test alone is insufficient to rule out CMV, its results are consistent with the findings from the CFA-based assessments.

The present study acknowledges that reliance solely on Harman’s single-factor test may not provide sufficient evidence to rule out common method bias (Podsakoff et al., 2003). Although the exploratory factor analysis indicated that a single factor did not account for the majority of variance, additional procedures were conducted to provide a more rigorous assessment.

Specifically, a marker-variable technique was employed in accordance with the procedure proposed by Lindell and Whitney (2001). A theoretically unrelated construct was designated as a marker variable, and partial correlation analyses were conducted to estimate the extent of potential method variance. After statistically controlling for the marker variable, the correlations among the primary constructs remained significant and substantively similar in magnitude.

These results provide additional evidence that common method variance does not substantially influence the observed relationships. Therefore, common method bias is unlikely to pose a serious threat to the validity of the findings reported in this study.

Taken together, the results from (a) the robust latent method factor analysis, (b) chi-square difference testing using appropriate scaling corrections, (c) comparisons with alternative measurement models, and (d) Harman’s single-factor diagnostic collectively indicate that common method variance does not represent a serious concern in the present study.

## 5 Discussion

### 5.1 General discussion

This study empirically examined the mechanisms through which perceived organizational virtue influences job performance by investigating the mediating role of LMX and the moderated mediation effect of PsyCap. The results showed that organizational virtue had a significant positive relationship with LMX, which, in turn, improved employees’ job performance. In addition, LMX mediated the positive relationship between organizational virtue and job performance, and PsyCap moderated the mediated relationship between organizational virtue and job performance via LMX. Specifically, the indirect effect of organizational virtue on job performance via LMX was stronger for those with higher levels of PsyCap.

The findings provide important insights into the role of PsyCap within the moderated mediation process. While organizational virtue consistently fostered high-quality LMX relationships, the extent to which such relational resources were translated into job performance varied depending on employees’ PsyCap. This suggests that PsyCap not only functions as a contextual factor shaping perceptions of organizational virtue or the formation of leader–member relationships, but also serves as a personal psychological resource that amplifies the effectiveness of LMX in increasing job performance. In addition, employees with high PsyCap are better able to leverage the trust, support, and mutual respect inherent in high-quality LMX relationships to enhance their job performance. On the other hand, employees with low PsyCap may experience positive LMX but, at the same time, may be less capable of converting these relational advantages into tangible performance gains. Thus, PsyCap functions as an important enhancer in the organizational virtue–LMX–job performance link, strengthening the indirect effect of organizational virtue on job performance through LMX.

### 5.2 Theoretical implications

This study provides several theoretical implications by clarifying the relational and psychological mechanisms through which organizational virtue contributes to employee performance outcomes.

First, this study advances the literature on organizational virtue by highlighting its relational implications for leader–member exchange (LMX). Prior research has primarily conceptualized organizational virtue as a cultural attribute that promotes ethical conduct, compassion, and positive organizational climates (Cameron et al., 2004). While such studies have emphasized the moral and emotional functions of virtuous organizational contexts, relatively limited attention has been paid to how these contexts influence the quality of leader–subordinate relationships. The findings of this study demonstrate that organizational virtue positively influences LMX, suggesting that a virtuous organizational culture not only promotes ethical values but also strengthens interpersonal trust and mutual respect within leader–member relationships. By empirically linking organizational virtue to LMX, this study extends existing research by showing that virtuous organizational environments function as an important relational foundation that facilitates high-quality leader–member exchanges.

Second, this study contributes to leadership and performance research by clarifying the mediating mechanism through which organizational virtue influences job performance (MacKinnon et al., 2012). Previous studies have consistently documented a positive relationship between LMX and employee performance (Gerstner and Day, 1997). However, the underlying contextual conditions that foster high-quality LMX have received comparatively less attention. The findings of this study indicate that LMX partially mediates the relationship between organizational virtue and job performance, suggesting that the performance-enhancing effects of virtuous organizational values are realized through relational processes between leaders and followers. By demonstrating that virtuous organizational contexts indirectly enhance performance through improved leader–member relationships, this study refines theoretical explanations of how ethical and virtuous organizational values can be translated into tangible performance outcomes.

Third, this study extends the literature on psychological capital by identifying its contingent role in the relational mechanism linking organizational virtue, LMX, and job performance. Psychological capital has been widely recognized as a positive psychological resource that enhances employee attitudes and performance (Luthans et al., 2007b; van Tonder et al., 2024). However, prior research has largely examined PsyCap as a direct predictor of performance-related outcomes (Luthans et al., 2006). The present study contributes to this literature by demonstrating that PsyCap moderates the mediating process through which organizational virtue influences job performance via LMX. The findings suggest that the indirect effect of organizational virtue on job performance through LMX becomes stronger when employees possess higher levels of psychological capital. This result highlights that PsyCap functions as a critical psychological resource that amplifies the effectiveness of relational mechanisms within organizational contexts.

Finally, this study contributes to the broader literature on positive organizational behavior and positive organizational scholarship by integrating organizational virtue, LMX, PsyCap, and job performance into a comprehensive conceptual framework. Previous studies in positive organizational scholarship have often focused on specific virtuous constructs such as compassion and their effects on employee outcomes (e.g., Choi and Ko, 2024; Ko and Choi, 2019; Rhee et al., 2017). In contrast, the present study adopts a broader conceptualization of organizational virtue and systematically examines how virtuous organizational values influence employee performance through both relational (LMX) and psychological (PsyCap) mechanisms. By doing so, this study advances theoretical discussions within the field by demonstrating how collective virtuous values embedded in organizational systems contribute to sustainable performance outcomes.

### 5.3 Practical implications

This study provides several practical implications for organizations seeking to enhance sustainable performance through positive organizational environments and leadership practices.

First, the findings suggest that cultivating organizational virtue can serve as an important foundation for improving leadership practices within organizations. The present study demonstrates that organizational virtue positively influences leader–member exchange (LMX), indicating that organizational environments characterized by fairness, respect, and trust facilitate higher-quality relationships between leaders and their subordinates. From a managerial perspective, this implies that organizations should not view virtue solely as a moral or ethical value but as a strategic resource that shapes leadership effectiveness. Accordingly, organizations may consider incorporating virtuous values into leadership development programs, ethical guidelines, and organizational culture initiatives in order to foster relational trust within teams.

Second, the results highlight the managerial importance of strengthening high-quality LMX relationships as a mechanism for improving employee performance. Consistent with the relational leadership perspective, the findings indicate that the quality of interactions between leaders and subordinates plays a critical role in translating organizational values into individual performance outcomes. Thus, organizations should emphasize relational leadership practices such as transparent communication, supportive supervision, and fair decision-making processes. Leadership training programs that enhance relational competencies may therefore contribute to improved employee performance and long-term organizational effectiveness.

Third, the findings underscore the strategic importance of developing employees’ psychological capital (PsyCap) as a positive psychological resource within organizations (Larson and Luthans, 2006; Ullah et al., 2024). The results demonstrate that PsyCap strengthens the indirect relationship between organizational virtue and job performance through LMX, suggesting that employees with higher levels of hope, resilience, efficacy, and optimism are better able to leverage positive relational environments. Human resource practitioners may therefore design systematic developmental programs—including coaching, training workshops, and psychological resource development initiatives—to enhance employees’ PsyCap. Such interventions can strengthen employees’ internal capacities to respond positively to relational leadership environments.

Fourth, the findings imply that organizations should adopt an integrated management approach that simultaneously promotes organizational virtue, relational leadership practices, and psychological resource development. Rather than relying exclusively on short-term performance incentives, organizations may benefit from establishing sustainable management systems that encourage ethical organizational cultures, high-quality leader–member relationships, and the development of positive psychological resources. Through this integrated approach, organizations can foster mutually reinforcing dynamics in which virtuous organizational environments enhance relational quality and employee performance, ultimately contributing to long-term organizational sustainability.

### 5.4 Limitations and future research directions

Despite its contributions, this study has several limitations that should be acknowledged, which in turn provide directions for future research.

Before discussing the more specific methodological limitations of the present study, it is important to consider several alternative explanations that may also account for the relationships observed in this study. Although the findings suggest that organizational virtue contributes to job performance through relational and psychological mechanisms, it is possible that other organizational factors may simultaneously influence these relationships. For example, supportive leadership styles, organizational support systems, or human resource management practices may foster both high-quality leader–member exchange relationships and higher levels of employee performance. In such cases, the observed relationships between organizational virtue, LMX, and job performance may partly reflect broader organizational conditions that encourage positive employee attitudes and behaviors. Future research may therefore benefit from incorporating additional organizational-level variables to more comprehensively examine the mechanisms through which virtuous organizational environments influence employee performance.

In addition, the cultural context of South Korea may also shape the relationships observed in this study. South Korean organizations are often characterized by relatively strong collectivist cultural values and hierarchical relationship structures, which may influence how employees perceive leadership relationships and organizational norms. In collectivist cultural contexts, employees may place greater emphasis on relational harmony, mutual obligations, and respect for authority, which could strengthen the role of leader–member exchange as a key mechanism linking organizational virtue to employee performance. At the same time, hierarchical organizational cultures may influence employees to evaluate leadership relationships and organizational values in ways that differ from those observed in more individualistic contexts. These contextual characteristics should therefore be considered when interpreting the findings of this study.

Beyond these contextual considerations, several methodological limitations should also be acknowledged.

First, the generalizability of the findings may be constrained by the characteristics of the sample. The respondents were primarily drawn from a specific geographic region and consisted largely of employees in regular employment positions with relatively high educational backgrounds. These characteristics may limit the extent to which the findings can be generalized across diverse organizational contexts. Future studies should attempt to collect more heterogeneous samples across different industries, employment types, and organizational levels. Additionally, examining whether the relationships identified in this study vary across demographic factors such as gender, employment status, or organizational tenure may provide further insights.

Second, this study utilized a cross-sectional research design, which limits the ability to draw definitive causal conclusions among organizational virtue, LMX, PsyCap, and job performance. Because the data were collected at a single point in time, it is difficult to fully capture the dynamic processes through which organizational values influence relational and psychological mechanisms. Future research should consider employing longitudinal designs or experimental approaches to more rigorously examine the causal relationships among these variables.

Third, because the data were collected from organizational members in South Korea, caution should be exercised when generalizing the findings to other cultural and institutional contexts. Future research may therefore benefit from testing the proposed research model across diverse cultural environments in order to examine the robustness and cross-cultural applicability of the relationships identified in this study.

Fourth, while this study focused on the moderating role of psychological capital within the mediated relationship between organizational virtue, LMX, and job performance, future research may extend the model by incorporating additional positive psychological or emotional constructs. The growing literature on positive organizational scholarship suggests that variables such as positive emotions, positive organizational identity, and positive work-related identity may also play important roles in shaping organizational outcomes (Ko and Choi, 2021). Future studies may therefore expand the current framework by examining how various positive psychological resources interact with organizational virtue and leadership relationships to influence employee performance.

## Data Availability

The raw data supporting the conclusions of this article will be made available by the authors, without undue reservation.
